# Detectability of simulated apical lesions on mandibular premolars and molars between radiographic intraoral and cone-beam computed tomography images: an ex vivo study

**DOI:** 10.1038/s41598-022-18289-3

**Published:** 2022-08-18

**Authors:** Thomas Gerhard Wolf, Fernando Castañeda-López, Lisa Gleißner, Ralf Schulze, Robert Kuchen, Benjamín Briseño-Marroquín

**Affiliations:** 1 Department of Restorative, Preventive and Pediatric Dentistry School of Dental Medicine, University of Bern, Freiburgstrasse 7, 3010 Bern, Switzerland; 2 Private Practice, San Luis Potosí, Mexico; 3 Department of Periodontology and Operative Dentistry, University Medical Center of the Johannes Gutenberg-University, Mainz, Germany; 4 Department of Oral and Maxillofacial Surgery, University Medical Center of the Johannes Gutenberg-University, Mainz, Germany; 5 Division of Oral Diagnostic Science, Department of Oral Surgery and Stomatology, University of Bern, Bern, Switzerland; 6 Institute for Medical Biometrics, Epidemiology and Informatics, University Medical Center, Johannes Gutenberg University Mainz, Mainz, Germany

**Keywords:** Oral anatomy, Outcomes research, Biological physics, X-rays

## Abstract

Adequate endodontic diagnostic is essential when making a therapy decision. Radiographic imagining acquisition methods (IAMs) are fundamental apical lesions of endodontic (ALE) origin diagnose tool. Thus, the aim of this research was to compare the simulated apical lesions (SALs) diagnose potential of digital intraoral radiography (DIR) and cone-beam computed tomography (CBCT), if there is a relationship between the IAMs, SALs-depth and their correct diagnose likelihood in human mandibular specimens’ datasets. 1024 SALs were prepared in cancellous and cortical bone with different penetration depths. The SALs-stages were radiographed with CBCT and DIR. The IAMs were randomly evaluated by 16 observers in two trials. Possible SAL findings were analyzed according to a five-point scale. The null hypothesis established that SALs detection accuracy does not differ between CBCT and DIR. Significantly differences (first 0.935 and second trial 0.960) were found for the CBCT area under the curve when compared with the DIR (first 0.859 and second trial 0.862) findings. SALs of smaller size were earlier detected by CBCT. In SALs without cortical involvement the probability of detection increased from 90 to 100%. The SALs-depth had the highest detectability influence on cancellous bone lesions and CBCT SALs detectability was 84.9% higher than with DIR images. The CBCT diagnose reproducibility was higher than the one of DIR (Kappa CBCT 75.7–81.4%; DIR 53.4–57.1%). Our results showed that CBCT has a higher SALs IAM diagnosing accuracy and that SALs detection accuracy incremented as the SALs-size increased.

## Introduction

Adequate endodontic diagnostic of apical/periodontal lesions of endodontic (ALE) origin is compulsory so that the operator can make a therapy decision. In addition to the oral tissues examination, radiographic imagining acquisition methods (IAM) could be considered as a powerful endodontic ALE diagnose tool. Two-dimensional conventional intraoral (CIR) or digital intraoral radiography (DIR) are the most commonly endodontic IAMs employed^1,2^. Yet, these IAMs can display the anatomical structures only in a two-dimensional plane. Furthermore, anatomical structures superimposition may occur, complicating the area of interest assessment^3^ and a standardizable projection geometry is hardly accomplished^4^. However, intraoral dental images are capable of reproducing minute details^5^.

A DIR image is obtained on a photosensitive image receiver (detector), the detector converts the intensities proportionally into charges, which are displayed pixel by pixel on a monitor^5^. Different three-dimensional IAMs such as tuned aperture computed tomography, magnetic resonance imaging and multidetector computed tomography, due to either a low resolution, high acquisition cost or radiation dose and lack of availability as dental equipment have shown to have little value for endodontic diagnostics^3,5^. CBCT, on the contrary, due to its lower effective radiation dose^6,7^, appropriate image dental hard tissues differentiation^8^, ease of clinical use and small footprint, its use is considered in the meantime as a routine three-dimensional IAM in dental practice^1,2,9^ and has experienced an increasing use in endodontic diagnose^10^; yet, it should be stressed that with CBCT, metallic structures lead to artifacts^5^. CBCT^6,7^ is an IAM in which a large number of individual images are taken around the object and whose geometric arrangement is exactly known^5^. A computer, based on the images obtained, assumes a three-dimensional voxel grid and an image is created and displayed in axial, sagittal and coronal planes^5^. Being a calculated dataset, it is impossible to errorless reconstruct the object reality, thus, the dataset should be regarded as a “good estimate”^5^. CBCT may be more suitable for endodontic diagnosis when spatial orientation is essential and the diagnose problem justifies the higher radiation dose.

The radiological identification/diagnose of simulated apical lesions (SALs) has been differently investigated. Different research groups^11,12^ have reported that SALs that cancellous bone lesions are more difficult to be recognized with CIR. This is in contrast with reports^13,14^, in which SALs within the cancellous bone were radiologically recognizable. Different researchers^12,15^ were not able to observe a SAL detectability difference between DIR and CIR. Contrasting, it has been reported^16^ that in cases of a non-existent maxillary or mandibular SAL, CIR had higher diagnostic accuracy; whereas, when SALs involve cancellous and cortical bone then DIR had a higher detectability. Furthermore, it has also been reported^17,18^ that DIR contrast and brightness manipulation, enhances SALs detection; yet, that monitor color manipulation does not enhance their recognizability^15^.

### Study aim

Thus, the aim of this research was to investigate if CBCT and DIR differ regarding detection accuracy of SALs and if a correlation between SAL-depth and diagnostic accuracy can be observed. In this study, it is hypothesized that SALs can be diagnosed with the CBCT and DIR image acquisition methods (IAMs).

## Materials and methods

This research conducted with a radiological human mandibular dataset from a former collection from a dental school of a Mexican university that consisted of five human mandibular specimens containing intact teeth and without soft tissue. Prior to any experimental procedure the mandibles were radiographed with a digital panoramic X-ray device (Orthophos SL; Sirona Dental Systems GmbH, Bensheim, Germany/63 kV, 63 kV, 8 mA, 14 s.) Selection criteria were specimens in which no foreign objects (screws and plates), root canal treatment, or periapical pathosis were observed.

**Figure 1:**
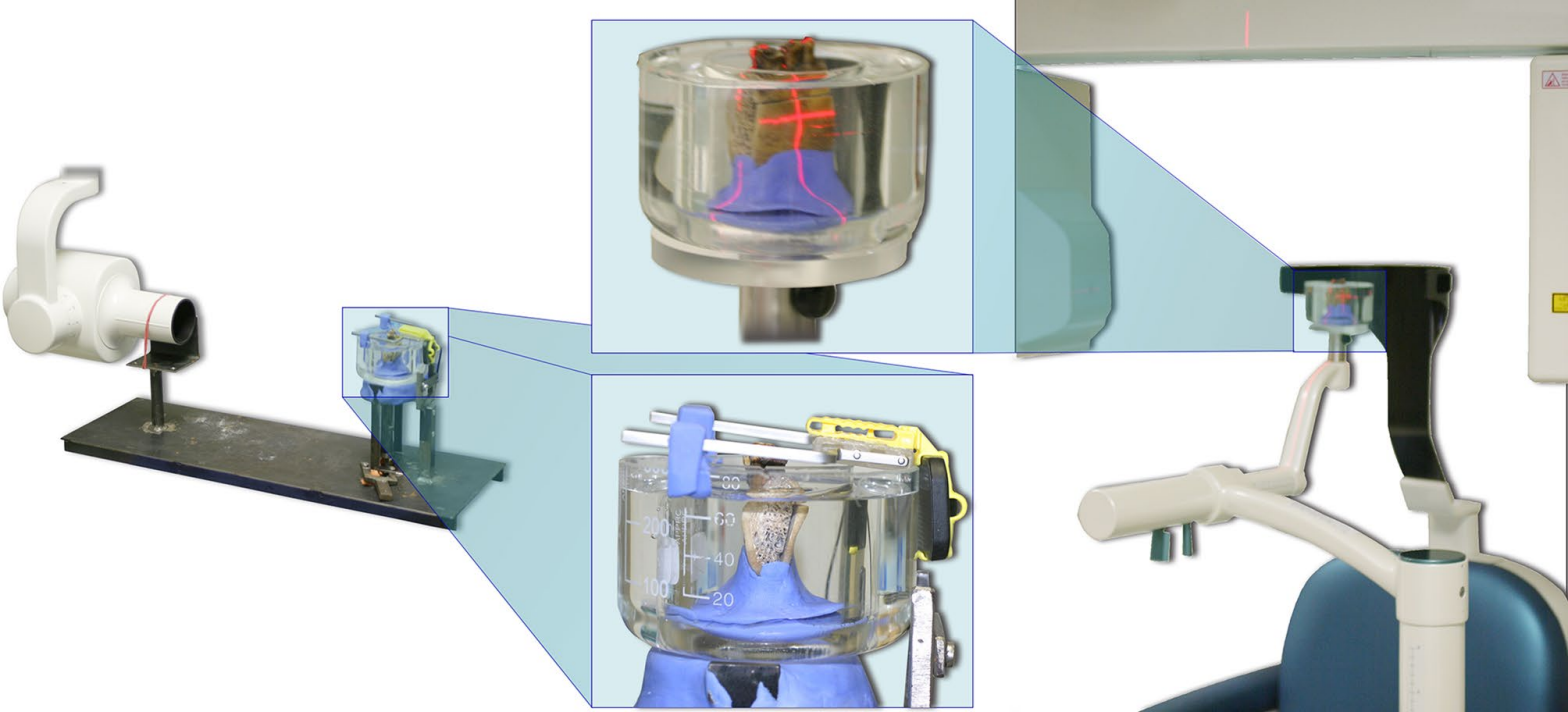
Reproduction of the radiological distance, angulation, soft tissue simulation and position of the specimens during exposition with DIR (left; distance between X-ray source to object: 480 mm) and CBCT (right) at all times.

**Figure 2:**
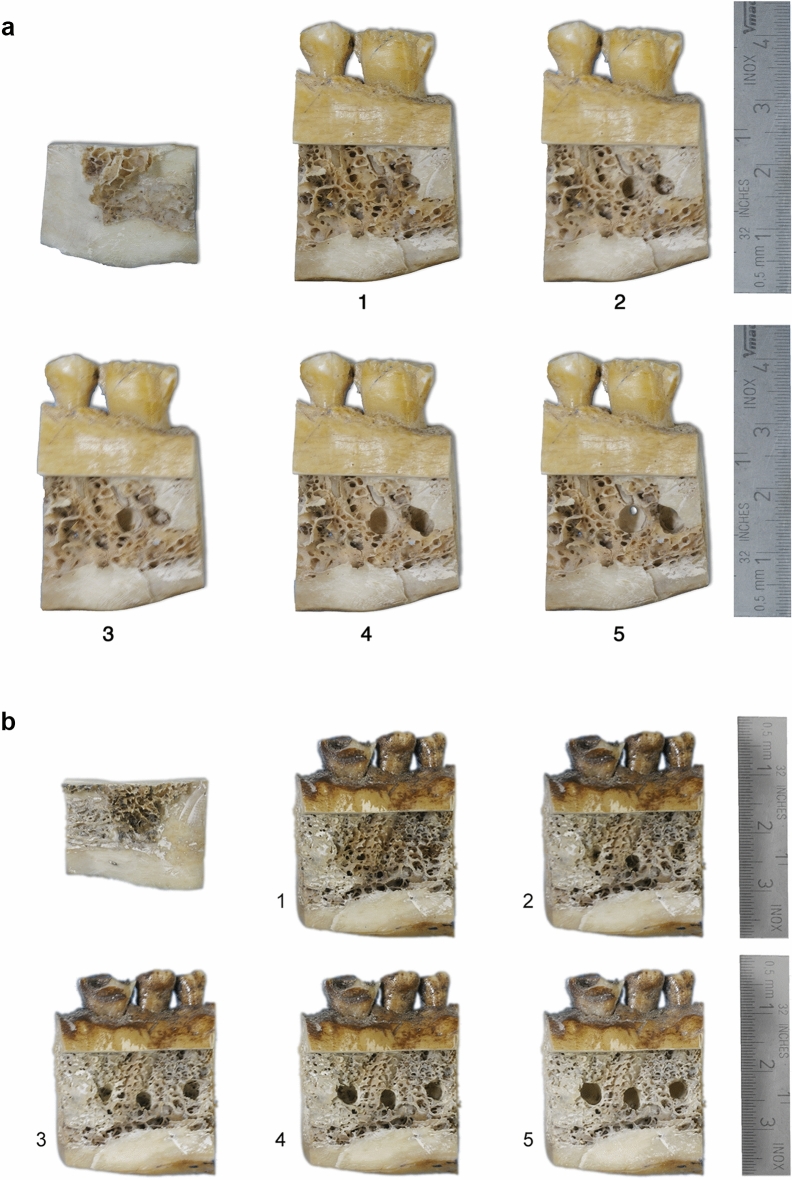
(**a**) Access to the apical region obtained through the design of a cortical plate (upper left) that could be repositioned without any interference. The SALs preparation at different stages (Table 1; 1 = no lesion; 2 = 50% of the bur diameter in cancellous bone; 3 = 100% of the bur diameter in cancellous bone; 4 = complete cancellous bone width; 5 = cortical bone perforated) can be observed from specimens one (no lesion) to five. (**b**) Access to the apical region obtained through the design of a cortical plate (upper left) that could be repositioned without any interference. The SALs preparation at different stages (Table 1; 1 = no lesion; 2 = 30% of the bur diameter in cancellous bone; 3 = 50% of the bur diameter in cancellous bone; 4 = 100% of the bur diameter in cancellous bone; 5 = complete cancellous bone width) can be observed from specimens one (no lesion) to five.

Eight blocks/specimens were obtained after sectioning the five mandibles. Each specimen had two teeth, either two premolars or molars and one premolar and molar with a total of 22 apical areas (AAs)/roots; in 16 of them a simulated apical lesion (SAL) was prepared. Silicone (Silagum Putty; DMG Chemisch-Pharmazeutische Fabrik GmbH, Hamburg, Germany) molded fixations were adapted to each specimen to be able to reproduce radiological exposition parameters (distance, angulation and position) of the specimens at all times. The distances of the CBCT were: X-ray-source to object-distance: 500 mm; X-ray source-image receptor distance: 799 mm and object-image receptor distance: 299 mm. The DIR distance between X-ray source to object was of 480 mm. Radiological soft tissue attenuation and scattering was simulated through water submersion of the in silicone previously fixed specimens (Fig. 1). Cone-beam computer tomography (CBCT) images were obtained with a 3D Accuitomo 80 (J. Morita Mfg. Corp., Kyoto, Japan) at 80 kV, 4 mA, a 60 × 60 mm field of view, and voxel size of 125 µm. The digital intraoral radiographs (DIR) were taken with a Siemens Heliodent MD X-ray unit at 60 kV, 7 mA and 80 ms with a physical pixel size 0.0195 mm edge length CCD “Full Size Sensor” (Sirona Dental Systems GmbH, Bensheim, Germany) (Fig. 1). Access to the apical region bone was obtained designing a cortical plate created with an incision on the base and lingual aspects of the mandibles and then lifted taking care that they would not compromise the AA anatomy (Fig. 2a,b). With the cortical repositioned a CBCT and DIR individual control images were taken being considered as the “no lesion” group.

**Figure 3:**
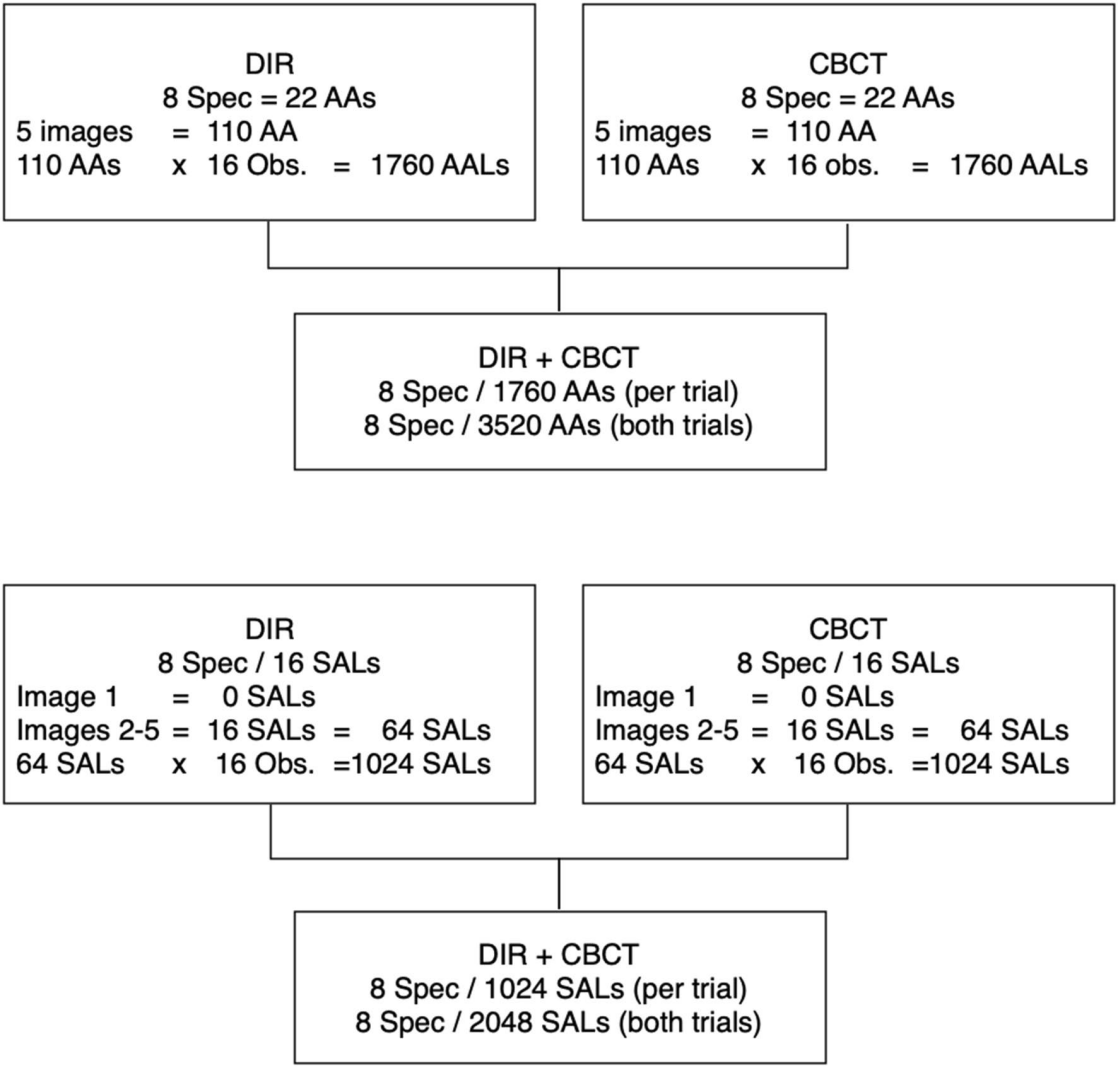
Flow diagram of data of all teeth/roots of the specimens (Spec.) to be diagnosed by means of digital intraoral radiographs (DIR) and cone-beam computed tomography (CBCT) by 16 observers (Obs.) in two observation trials with a 4-week difference time when considering the apical areas (AAs; AALs: apical area lesions) and simulated apical lesions (SALs). Specimen, AAs and Obs. total number determined to be diagnosed with DIR and CBCT (upper part). Specimen, SALs and Obs. total number determined to be diagnosed with DIR and CBCT (lower part).

All SALs were drilled (Fig. 2a,b) in randomly pre-selected AAs*,* in a bucco-lingual direction and under magnification (20×; Leica MZ6/Leica Microsystems, Wetzlar, Germany). The first SAL was superficially prepared in cancellous bone by means of a 012 (1.2 mm Ø) round bur (Hager & Meisinger GmbH, Neuss, Germany); it was meant to define the SAL location; thus, it was not evaluated. The next SALs were prepared in the same area with a diamond yellow-ringed round 014 bur (1.4 mm Ø; Horico-Hopf, Ringleb & Co. GmbH & CIE, Berlin, Germany) to different deepness of the cancellous and/or cortical bone (Table 1; Fig. 3); yet, without modifying their diameter (Fig. 2a,b). CBCT and DIR images with the repositioned cortical cortical plate were taken after the preparation of each SAL-stage was completed. The SAL-stages were radiologically (CBCT and DIR), photographically documented and electronically stored on a Fujitsu Siemens Esprimo PC (Fujitsu Technology Solutions GmbH, Munich, Germany).

**Table 1:** Simulated apical lesions (SAL) stages at which the specimens were X-rayed (CBCT and DIR) and photographed (bur diameter ± 0.02 mm; * = this SAL-stage was prepared only for the purpose of defining the SAL location, thus, not evaluated).

SAL	Lesion	SAL size (mm Ø)/limits
1st	No lesion	–
*	Superficial cancellous bone	0.3
2nd	50% of the bur diameter in cancellous bone	0.7
3rd	100% of the bur diameter in cancellous bone	1.4
4th	Complete cancellous bone width	Cancellous cortical limit
5th	Cortical bone perforated	Visible perforation

16 experienced observers, from different dental specialties (endodontics, periodontics, restorative dentistry, dental prosthodontics and oral surgery) with a minimum 4 years professional constant CBCT and radiographic diagnosis experience and previously calibrated (by means of an acquired research image set; however, not evaluated) diagnosed the DIR and CBCT images in two different observation trials with a time interval of 4 weeks. A total of 40 CBCTs and DIRs images were diagnosed by each observer in each trial. The IAMs obtained were randomized by means of the Research Randomizer software (4.0; Geoffrey C. Urbaniak and Scott Plous; 2013; downloaded on January, 2018, http://www.randomizer.org/). The first CBCT and DIR images of each specimen had no SAL and served as control group. The other four images of each specimen had at least one SAL. The radiological trace of the lifted cortical plates were disclosed to the observers prior to any diagnose procedure. The observers were prompted to diagnose the “presence” or “absence” of a SAL by means of a five-point confidence-scale: 1 = definitely present; 2 = probably present; 3 = uncertain; 4 = probably not present and 5 = definitely not present. The 1 and 2 scale points were considered as positive; whereas the 3, 4, and 5 scale points were considered as negative SALs diagnose. For the one and two scale points by means of CBCT, the observers were requested to set the corresponding crosshairs at the intersection of the three planes in the center of the SAL, thus, enhancing verification of a targeted SAL. No information was given concerning the AA location or specimen SALs number. The diagnose procedures were done with a Samsung SyncMaster S24A650D LED-blacklit LCD monitor (Samsung Electronics Co., Ltd., Samsung Group, Seoul, South Korea; 1920 × 1080 pixels; 27 inches; brightness = 300 cd/m^2^; contrast = 5000:1). Prior to each diagnostic session, the monitor was calibrated (German standard^19^; TG18-0IQ). The findings were protocoled by a study non-participating individual. The DIR images were evaluated as IrfanView TIFF files (IrfanView 4.42-64 Bit 1996; Irfan Skiljan, Vienna, Austria) being the observers allowed to modify the gray values. The CBCT images were evaluated with the One Data Viewer Plus (J.Morita MFG, CORP, Kyoto, Japan) software being the observers allowed to scroll through the x, y, and z slices and to adjust the gray values.

### Statistical analysis

Statistical significance (p ≤ 0.05) was assessed regarding the null hypothesis which established that SALs detection accuracy does not differ between CBCT and DIR. Four probable results, based on the five points diagnose scale (Table 1), were evaluated: SAL correctly diagnosed (1, true-positive), present but not diagnosed (2, false-negative), not present and as such diagnosed (3, true-negative), and not present but diagnosed (4, false-positive). The sensitivity and specificity were analyzed for pooled observers. Receiver operating characteristic (ROC) curves were calculated for each IAM and observation trial for all 16 observers to assess the diagnostic accuracy of the IAMs. The optimal threshold values for differentiation were calculated with the Youden index (sensitivity + specificity)—1. The inter-rater reliability agreement of the results of the observers pooled were determined with the Fleiss’ Kappa (suitable for more than two observers) for each IAM (individually and combined) and observation trial. Kappa agreement weighting was determined according to the Landis and Koch guideline values. If the covariates IAM, SAL-depths and observation trial were associated with the probability of a correct diagnose (correctly diagnosed [0] and incorrectly diagnosed [1]) was determined with a binary logistic regression. The omnibus test of model coefficients allowed the independent variables (IAM, SAL-depth and observation trial) to be included in the model, thus, to evaluate the influence of low or high (> 0.05) significances. The model quality was assessed with the Cox and Snell’s r-square and its adjusted version (Nagelkerke’s r-square). The data was analyzed with the IBM SPSS Statistics data editor (SPSS 23.0.0.0 for Windows, SPSS, Chicago, Ill., USA). The statistical methods used were validated by the Institute of Medical Biometry, Epidemiology and Informatics (IMBEI; University Medical Center, Mainz, Germany).

### Ethical approval

No further ethical approval was required since this research was conducted with a radiological human mandibular dataset from a former, non-archeological, collection from a dental school of a Mexican university.

## Results

**Figure 4:**
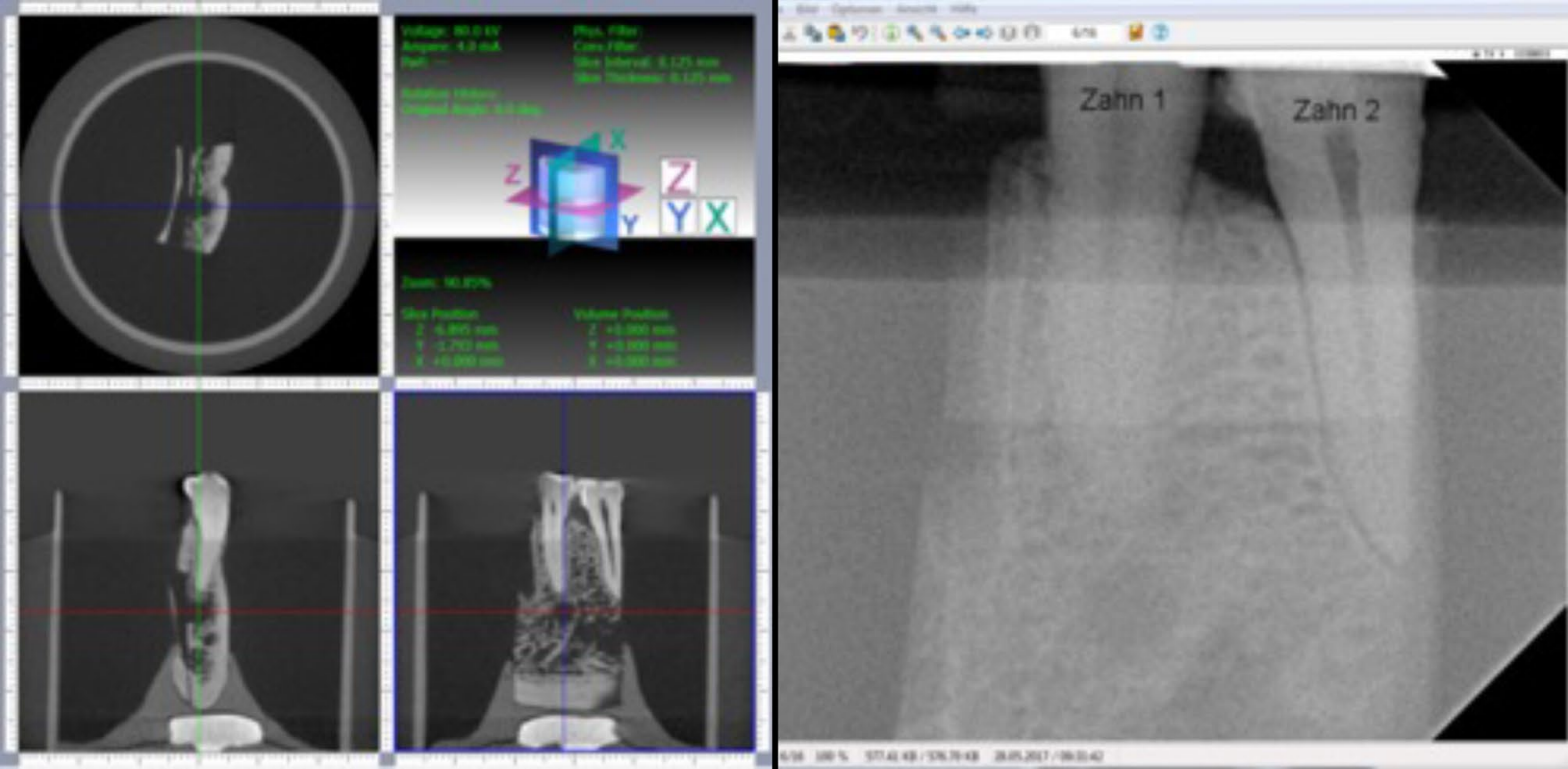
Screenshots of the working areas with CBCT (left) and DIR (right) with the same research specimen. The SAL observed correspond to a stage 5 (cortical bone perforated). On the DIR image it can be observed that the incision on the base and lingual aspects of the mandibles did not compromise the AA anatomy.

22 apical areas (AA)/roots were observed in the eight sectioned specimens. In 16 AAs a simulated apical lesion (SAL) was prepared. One cone-beam computed tomography (CBCT) and digital intraoral radiograph (DIR) image was made at each SAL-stage (Table 1; Figs. 3, 4). Thus, 1760 AAs were assessed by means of CBCT or DIR images by 16 observers in each trial. Of the 1760 AAs 1280 had a SAL. Thus, 1024 radiologically existent SALs were diagnosed with both image acquisition methods (IAMs). Two different observation trials were carried out with a 4-week time difference.

**Figure 5:**
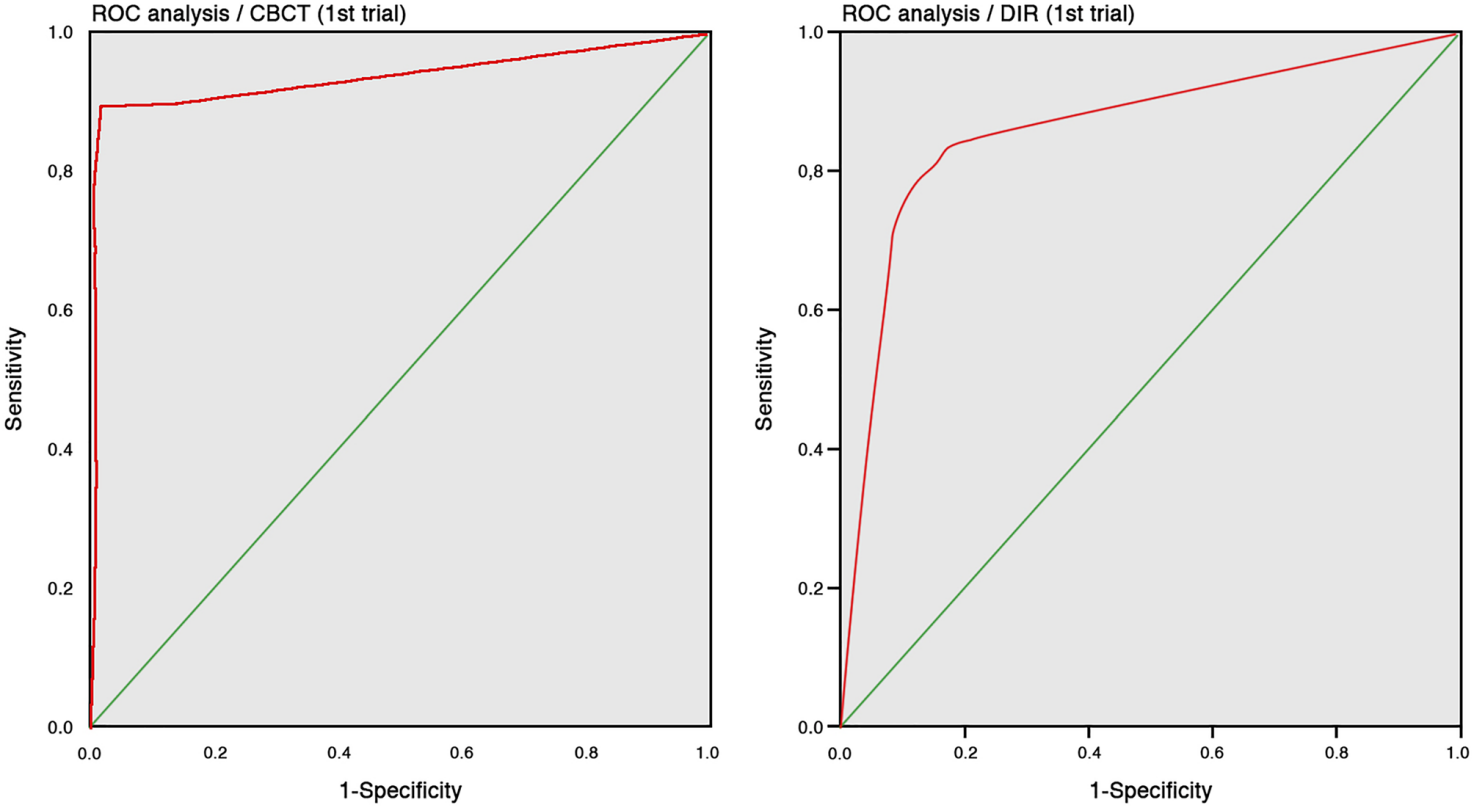
ROC analysis: CBCT (left) and DIR (right) of the first trial being the16 observers pooled with both image acquisition methods.

**Figure 6:**
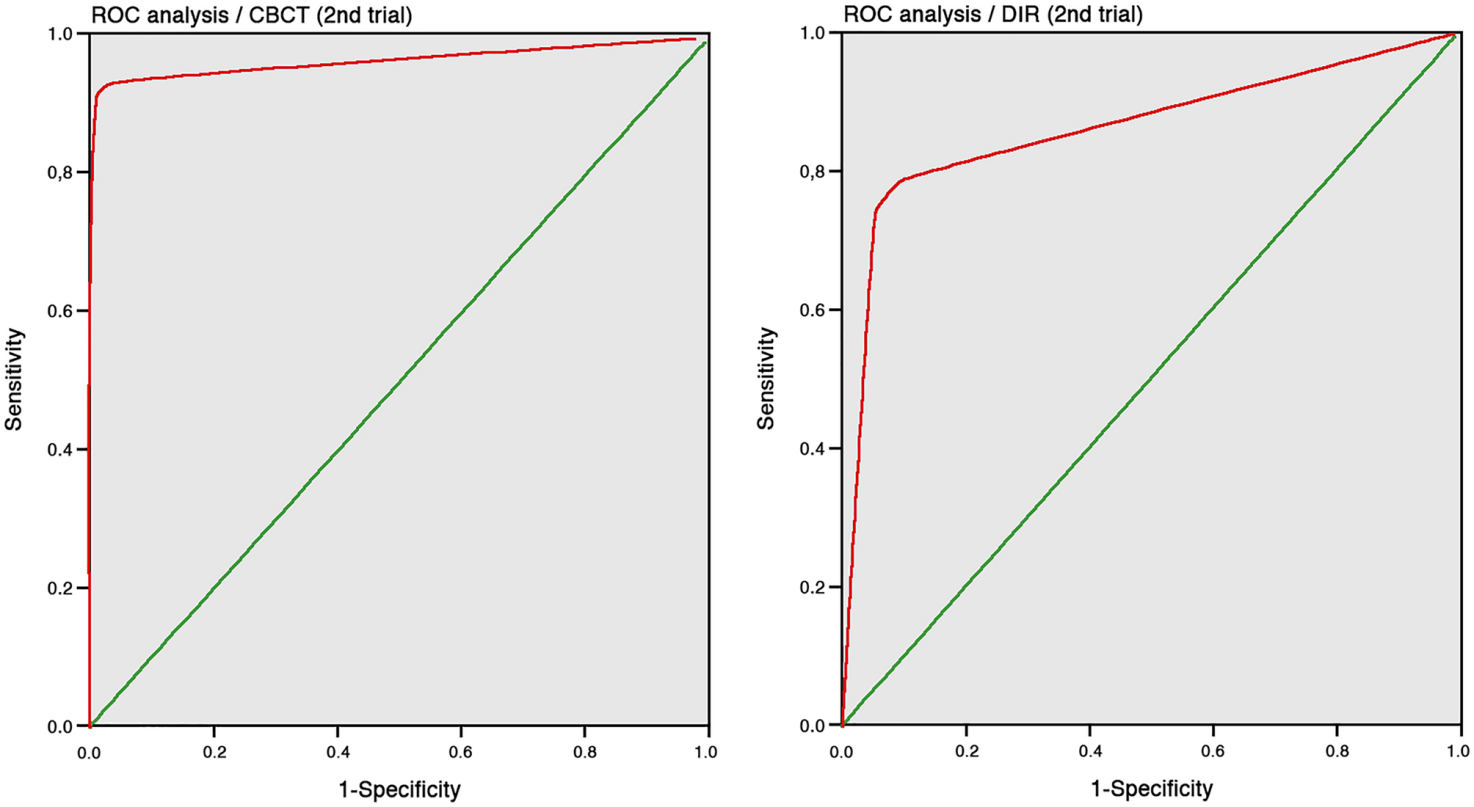
ROC analysis: CBCT (left) and DIR (right) of the second trial being the16 observers pooled with both image acquisition methods.

The CBCT and DIR images ROC findings of the first trial of pooled observers resulted in an AUC (area under the curve) of 0.935 and 0.859 (Fig. 5), respectively. The second trial findings resulted in an AUC of 0.960 and 0.862 (Fig. 6), respectively. All results are statistically significant (p < 0.001). The individual observer AUC values for the first and second trials of both IAMs are shown in Table 2. The sensitivity, specificity and optimal threshold of both IAMs are shown in Table 3.

**Table 2:** Individual AUC (area under the curve) values of the 16 observers for the first (1) and second (2) trials of the digital volume tomography (CBCT) and intraoral images (DIR) image acquisition methods.

Observer	CBCT/1	DIR/1	CBCT/2	DIR/2
1	0.963	0.912	0.910	0.810
2	1.000	0.833	0.932	0.845
3	0.914	0.895	0.866	0.843
4	0.903	0.939	1.000	0.916
5	0.982	0.840	0.938	0.819
6	0.906	0.872	0.938	0.875
7	0.932	0.916	1.000	0.829
8	1.000	0.913	1.000	0.924
9	0.982	0.896	1.000	0.921
10	0.903	0.863	0.919	0.782
11	0.965	0.883	0.949	0.834
12	0.903	0.900	0.998	0.909
13	0.896	0.778	1.000	0.808
14	0.906	0.887	1.000	0.903
15	0.906	0.815	0.906	0.906
16	0.950	0.709	1.000	0.870

**Table 3:** Statistic results of the16 observers pooled for digital volume tomography (CBCT) and intraoral images (DIR), first trial (1st) and second trials (2nd), respectively (AUC = area under the curve; α = 5%, p < 0.001; Youden index).

Method	CBCT/1	DIR/1	CBCT/2	DIR/2
AUC	0.935	0.859	0.960	0.862
Sensitivity	0.89	0.79	0.93	0.79
Specificity	0.96	0.87	0.98	0.91
Optimal threshold	0.85	0.66	0.91	0.70

The individual inter-rater IAMs reliability results, when comparing the two observation trials results of the same IAM and between the different IAMs in the same trial are shown in Table 4. In the first and second trials a CBCT and DIR inter-rater reliabilities of 83.7 and 67.6%. and 87.3 and 70.3% were observed, respectively. The CBCT the weighted agreement was considered as almost perfect in both trials, whereas the one of both trials within the DIR findings was considered as substantial.

**Table 4:** Inter-rater reliability for the digital volume tomography (CBCT) and intraoral images (DIR) of the first (1) and second (2) observation trials of the16 observers pooled (kappa = Kappa coefficient; lwr.ci and upr.ci = lower and upper bound of the 95% confidence interval, respectively).

	Kappa	lwr.ci	upr.ci
CBCT/1	0.84	0.81	0.86
CBCT/2	0.87	0.85	0.90
DIR/1	0.68	0.65	0.70
DIR/2	0.70	0.68	0.73
CBCT/1 and CBCT 2	0.88	0.83	0.92
DIR/1 and DIR/2	0.72	0.67	0.81
CBCT 1 and DIR/1	0.69	0.64	0.78
CBCT/2 and DIR/2	0.69	0.64	0.78

**Figure 7:**
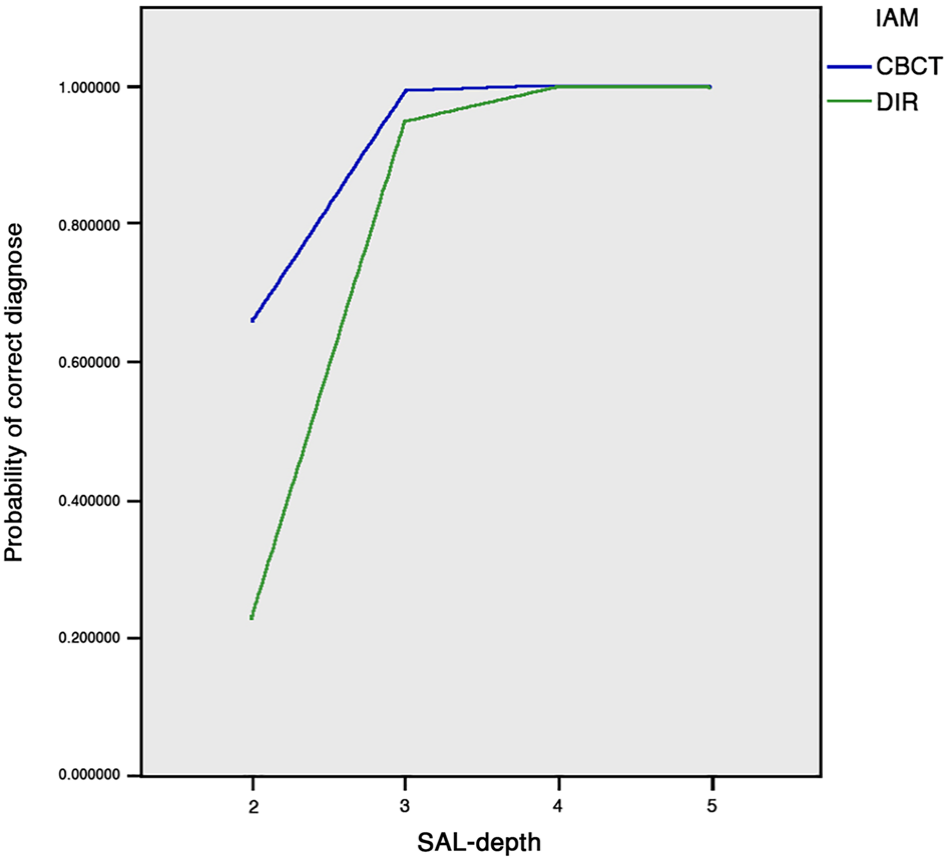
Relationship between image acquisition method (IAM), simulated apical lesion (SAL) depth, and SAL diagnose (2 = 50% of the bur diameter [0.7 mm Ø] in cancellous bone; 3 = 100% of the bur diameter in cancellous bone[1.4 mm Ø]; 4 = bur through cancellous bone up to cancellous cortical bone boundaries; 5 = cortical bone perforated [visible perforation]).

The covariates SAL-depth (correctly [1]; incorrectly diagnosed [0]) and IAM were both significantly associated with the probability of a correct diagnose (estimated parameters: 109.222 and 48.17, respectively. Both p-values < 0.001). The covariate observation trial was, however, not significantly associated with the probability of a correct diagnosis (estimated parameter: 0.833; p-value ca. 0.361). Furthermore, when the SAL-depth was incremented by one stage, the correct diagnose probability increased 100% and a correct DIR-SAL diagnose probability decreased by 84.9% when a CBCT diagnose was not available. The model qualities obtained were classified as a strong effect (0.380, Cox und Snell r-square; 0.670, Nagelkerke r-square, ƒ = 1.42, Cohen’s effect size conversion). Correlations between the SAL-depth as well as IAM and correct diagnose were observed. However, the SAL-depth had a higher influence on the correct diagnose than the IAM employed (Fig. 7).

## Discussion

The radiological identification of apical lesions of endodontic (ALE) origin has been reported with different image acquisition methods (IAMs). The present study investigated the digital intraoral radiograph (DIR) and cone-beam computed tomography (CBCT) potential to diagnose simulated apical lesions (SALs), the findings reproducibility, possible relationship between the IAMs and SALs-depth, and likelihood of a correct diagnose. The SAL detectability in maxillary specimens has been investigated^20–22^; however, maxillaries were not considered in this investigation due to the difficulty to standardize the SALs parameters according with the ones of this investigation. Furthermore, contrary to this investigation, when investigating maxillary specimens, the SALs preparation have to be initiated from the cortical to the cancellous bone or the teeth must be extracted and repositioned, thus, not mimicking a cancellous to cortical bone usual apical lesion evolution. The specimens in this research were designed taking care that the SALs-depth range was equally distributed and that the DIR and CBCT projection geometry were reproducible at all times. A possible disadvantage of this research could be that the number of diseases and non-diseased sites are uneven; however, this was due to the intrinsic limitation of the human dataset did not allow to consider this parameter in a different way.

Similar to different reports^15,17,18,23^ soft tissue absorption was achieved submerging the specimens in two-nested glass beakers filled with water. A room illuminance, placed behind the observer, increment to 50–80 lx is advantageous for diagnosing radiographs with a monitor when compared with complete darkness^24^; yet, such illuminance conditions are difficult to maintain in the daily practice. It has also been reported^25^ that ambient light (up 500 lx,) which is often recommended for dental offices, has no negative effect on radiographic diagnose. Thus, since the illuminance of the diagnostic room was perceptibly lower than 500 lx, no negative influence on diagnostic performance can be expected in the present study. Moreover, a constant diagnostic quality was maintained with a 1920 × 1080 pixels monitor matrix, compliant test image calibration prior to each observer evaluation^19^ and image (TIFF) quality preservation.

Although radiographic diagnosis is considered a subjective image interpretation which, due to a relatively low inter-observer agreement is generally difficult to reproduce^26^, several studies^2,15,27^ have been conducted with a discipline-limited and/or smaller observer population. Therefore 16 observers, from different dental specialties (endodontics, periodontics, restorative dentistry, dental prosthodontics and oral surgery), were implemented to prevent a low inter-observer agreement; furthermore, the disagreement degree was controlled through observer calibration (SALs DIR and CBCT images excluded from the study) and without providing any SALs information^28^. SALs have been chemically (70% perchloric acid) prepared^9,23^. The authors are of the opinion that this experimental set-up closely resembles an in-vivo osteolysis process. However, this methodology was not employed in this investigation, due to the controlling difficulties of the SALs localization, size and depth parameters.

Although, due to intrinsic research parameters, the SALs in this research could only be prepared in a buccal direction, an advantage of the parameters used in this investigation is that evolution of an apical lesion could be reproduced more closely to an in vivo situation. In different SALs investigations the lesions have been prepared with a bur directly on the cortical bone^11,12,18^. These type of SALs produce clear lesions boundaries, thus, being potentially easier to identify. Yet, this approach was not employed in this study since it is contradictory to an average ALE development. SALs have also been prepared by means of a bur on the alveolus after having extracted the corresponding tooth^12,16,20,29,30^. A drawback of this methodology is that an exact repositioning of the tooth is mandatory making it difficult to avoid a fissure development or damage to the apical tooth socket zone^31^ and that a progressive SAL expansion in cortical bone direction is not possible. Thus, in order not to disrupt this area, a cortical bone plate was lifted allowing direct cancellous and cortical bone drilling at the root apex area^13–15,17,27^. Care was taken that the cortical gap did not radiographically interfere with the SAL localization, thus. Moreover, the images with the cortical plate repositioned; yet, still without a SAL, were used to advert the observes where the separation gap was located.

The receiver operating characteristic (ROC) resulting areas under the curve (AUC) showed a higher (DIR = 0.859-first, and 0.862-s trial; CBCT = 0.935-first, and 0.960-s trial) CBCT-SAL diagnostic accuracy when compared to DIR. These results are in agreement with similar ones previously reported^27,32^. However, their observer numbers (two to six) are markedly lower than the one of this investigation (16). CBCT showed sensitivities of 89% (first) and 93% (second trial) when SALs were present and of 96% (first) and 98% (second trial) in specimens without SALs. These results are within the range of the ones previously reported^32^. A higher specificity than sensitivity in the first (specificity 0.87, sensitivity 0.79) and second trials (specificity 0.91, sensitivity 0.79) obtained with the DIR images proved a higher DIR accuracy in specimens without SALs. These results are consistent with a clinical^2^, a retrospective^33^ and ex-vivo studies with human specimens^27,30^. However, the relative high DIR sensitivity obtained, when comparing previous reports^13–15,17,27^ is noteworthy. This could be explained through the “idealized” SALs preparation with a consistent geometry with respect to the central ray and exposure reproduction methodology.

Dental CBCT-devices are known to produce different image quality when comparing different protocols within one device as well as between CBCT-devices^34^. Hence, it is difficult to extrapolate the results obtained to other settings or devices. However, as the Accuitomo-CBCT is a common device for clinical endodontics since it offers a small field of view and a high image quality^34^, thus, we are of the opinion that our results are relevant for the endodontic/dental community. The inter-rater agreement showed values between 83.7 and 87.3% for the CBCT and between 67.6 and 70.3% for the DIR methods in the first and second trials, respectively. Accordingly, the CBCT inter-rater strength of agreement was considered as almost perfect whereas the one of DIR was considered as substantial. The higher CBCT inter-rater reliability results are supported by the ones earlier reported^27^. Furthermore, the inter-rater reliability suggests also^31^ the benefit of a double IAM interpretation at different evaluating times. However, clinically seen, it would be difficult to routinely implement this suggestion, due to effective radiation dose and morphological factors.

The correlation between the IAM, SAL-depth, and SALs detectability and the detectability dependent variable in a total of 1024 SALs is well above the recommended one^35^. The fact that at the second and third SAL-depth stages (without cortical involvement; the probability of a correct diagnose was increased between 90 and 100% with each SAL-stage/increment, proved the radiological detectability of isolated cancellous bone lesions; Thus, the SALs-depth had the highest detectability influence on cancellous bone lesions. The CBCT SALs detectability was 84.9% higher than with DIR images, even at the lowest SAL-stage. However, in the second trial no IAM detectability influence was observed. Thus, when considering a clinical situation, CBCT seems to be the more suitable to detect existing ALE. Our results are supported by previous ex-vivo^30^ and in-vivo studies^2,21,22,36^; yet, with a maximum of six observers. Taking into account that every radiation exposure not performed represents a 100% dose saving for an individual^5^ a clinician should always bear in mind the possibility of an existing ALE, in cases where no lesion is expected (e.g. tooth with a vital pulp), it would be advisable to refrain from the use of a CBCT as a primary IAM. Thus, according to the observed SAL detectability potential of DIR and CBCT and taking into consideration that CBCT is a radiological method that exposes a patient to a higher effective dose when compared to DIR, it can be concluded that in cases where the presence of an ALE of endodontic origin is doubtful, that CBCT would be beneficial for the patient where the diagnose knowledge previously obtained with DIR or CIR could be enhanced and, most important, would have a positive treatment effect.

## Conclusions

CBCT seems to be the more accurate than DIR when diagnosing existing apical lesions of endodontic origin.

## Data Availability

The datasets used and/or analyzed during the current study available from the corresponding author on reasonable request.
